# Occupational exposure levels of bioaerosol components are associated with serum levels of the acute phase protein Serum Amyloid A in greenhouse workers

**DOI:** 10.1186/s12940-016-0090-7

**Published:** 2016-01-20

**Authors:** Anne Mette Madsen, Trine Thilsing, Jesper Bælum, Anne Helene Garde, Ulla Vogel

**Affiliations:** National Research Centre for the Working Environment, Lersø Parkallé 105, DK-2100 Copenhagen, Denmark; Research Unit for Occupational and Environmental Medicine, Institute of Clinical Research, University of Southern Denmark, DK-5000 Odense C, Denmark; Research Unit for General Practice, Department of Public Health, University of Southern Denmark, DK-5000 Odense C, Denmark; OPEN, Odense Patient Exploratory Network, Institute of Clinical Research, SDU, Odense C, Denmark

**Keywords:** Serum Amyloid A, C-reactive protein, Dust, Endotoxin, Exposure, β-glucan, Fungi, Inflammation, Occupational health

## Abstract

**Background:**

Occupational exposure to particles may be associated with increased inflammation of the airways. Animal experiments suggest that inhaled particles also induce a pulmonary acute phase response, leading to systemic circulation of acute phase proteins. Greenhouse workers are exposed to elevated levels of bioaerosols. The objective of this study is to assess whether greenhouse workers personal exposure to bioaerosol components was associated with serum levels of the acute phase proteins Serum Amyloid A (SAA) and C-reactive protein (CRP).

**Methods:**

SAA and CRP levels were determined in serum sampled repeatedly from 33 greenhouse workers. Blood was drawn repeatedly on Mondays and Thursdays during work weeks. Acute phase protein levels were compared to levels in a comparison group of 42 people and related to individual exposure levels to endotoxin, dust, bacteria, fungi and β-glucan.

**Results:**

Serum levels of SAA and CRP were not significantly different in greenhouse workers and a reference group, or on the two work days. In a mixed model, SAA levels were positively associated with endotoxin exposure levels (*p* = 0.0007). Results for fungi were not clear. CRP levels were positively associated with endotoxin exposures (*p* = 0.022). Furthermore, when workers were categorized into three groups based on SAA and CRP serum levels endotoxin exposure was highest in the group with the highest SAA levels and in the group with middle and highest CRP levels. SAA and CRP levels were elevated in workers with asthma.

**Conclusion:**

Greenhouse workers did not have elevated serum levels of SAA and CRP compared to a reference group. However, occupational exposure to endotoxin was positively associated with serum levels of the acute phase proteins SAA and CRP. Preventive measures to reduce endotoxin exposure may be beneficial.

## Background

Occupational exposure to bioaerosols may be associated with increased inflammation of the airways [1], respiratory symptoms [2, 3] and occupational particle exposure is associated with increased risk of cardiovascular disease [4]. Inhalation of particles promotes cardiovascular risk possibly through induction of a pulmonary acute phase response [5]. The acute phase response is a complex systemic response to e.g. bacterial infection, tissue injury, and trauma characterized by up- and down regulation of blood levels of a variety of proteins, termed acute phase proteins, such as C-reactive protein (CRP) and Serum Amyloid A (SAA) [6, 7]. Pulmonary exposure to various types of particles including dust from biofuel plants converting straw, diesel exhaust particles, pure carbon particles such as carbon black and TiO_2_ nanoparticles all induce a pulmonary acute phase response in mice [8, 9]. Similarly, inhalation of endotoxin induces a strong pulmonary acute phase response in mice [10]. SAA is directly implicated in atherosclerosis as virus-mediated overexpression of SAA in susceptible mice (ApoE−/− mice) promotes plaque progression [11]. In agreement with this observation, acute phase response and serum levels of both CRP and SAA are associated with future risk of cardiovascular disease in prospective epidemiological studies [12, 13]. Increased serum levels of CRP and SAA are also associated with airway inflammation [14], asthma [15] and chronic obstructive pulmonary disease [16] .

Workers in greenhouses are exposed to very different concentrations of the bioaerosol components endotoxin, fungi, β-glucan and bacteria dependent on plant species, work task and ages of plants [17–19]. The greenhouse environment is different from e.g. agricultural environments as greenhouse workers mainly handle growing plants in enclosed environments while agricultural workers may work with animals or in open fields. In studies of bioaerosols and related health effects focus has mainly been on agriculture.

Occupational exposure to endotoxin is associated with airway symptoms of workers on straw and wood chips power plants [2], nasal inflammation of agricultural workers [20] and lung function decline of poultry workers [21]. Only few studies have documented occupational health effects from other bioaerosol components than endotoxin. However occupational exposure to airborne fungi and/or β-glucan of recycling workers [22] and farmers [23] and weight of dust for poultry workers [21] have been related to respiratory symptoms and/or inflammation. We have conducted a study with greenhouse workers where exposures to bioaerosols and serum levels of SAA and CRP were measured. Measures were done repeatedly during different seasons and on workers in three companies to cover different exposure levels. The aim of the current study was to determine whether occupational exposure of greenhouse workers to inhalable bioaerosol components is associated with increased serum levels of the acute phase proteins CRP and SAA. Since occupational exposure may occur continuously during a 40 year working life, occupationally associated increases in acute phase protein levels may be associated with increased risks related to inflammation mediated diseases such as asthma and cardiovascular disease.

## Methods

The study was approved by the Regional Ethics Committee of Southern Denmark (S-20100045) and the Danish Data Protection Agency of Southern Denmark (RSD: 2008-58-0035).

### Study group

A study was performed in three companies abbreviated A, B and C. Company A mainly produced and packed tomatoes, company B produced and packed cucumbers and company C produced and packed different potted flowering plants. Work in company A and B was performed in modern greenhouses or packaging halls; work in company C was mainly performed in modern greenhouses and some workers also worked some hours outdoor. The study was performed over a 2.5 year period (June 2010-December 2012). A total of 33 workers participated in the study; 11 from company A, eight from company B and 14 from company C. Asthma was defined as an affirmative answer to the question “Do you have asthma?”.

In addition, 42 participants from a population-based study on asthma and chronic rhinosinusitis (crs) [24] were included as a reference group. All controls were matched to the greenhouse workers according to sex, age and smoking status. The reference group comprised 33 individuals without self-reported symptoms of asthma and/or chronic rhinosinusitis (crs), and nine individuals with self-reported symptoms of asthma and/or crs as inflammatory airway diseases may affect the level of the acute phase proteins. Asthma was defined as reporting having ever had asthma and reporting at least one of the following symptoms in the last 12 months (i) wheeze or whistling in the chest, (ii) waking up with chest tightness, (iii) waking with shortness of breath, or (iv) waking with an attack of coughing [25]. Table 1 shows relevant demographic data.

**Table 1:** Characteristics of the study group of greenhouse workers and reference group

	Unit	Greenhouse workers		Reference group	
		All persons	With asthma^a^	All persons	With asthma^a^
Individuals	number	33	5	42	9
Age, mean, range	years	37.5	43.2	39.9	42.0
		20–57	28–54	15–57	29–57
Male	%	64	40	62	56
Current smoker	%	24	0	24	44
Asthma	%	15	100	21	100
Nasal allergy	%	24	80	36	78
Atopy	%	45	80	45	89
SAA^c^, average (median), range	mg/l	28.3	43.3	30.0	34.8
		(15.2)	(34.8)	(20.0)	(20.8)
		1.5–159	15.4–115	3.6–134	3.9–110
CRP, Average (median) range	mg/l	1.60	3.91	1.73	2.78
		(0.95)	(3.32)	(0.88)	(1.52)
		bd-13.3^b^	0.62–12.3	bd-8.1	0.35–8.1

^a^individuals with asthma and some also with chronic rhinosinusitis (crs)

^b^bd: below detection limit, ^c^average and medians of 166 SAA and 217 CRP samples

Atopy was defined by minimum 1 positive skin prick test (wheal diameter >3 mm) using standard allergens. Nasal allergy was in both cohorts defined by an affirmative answer to the question “Do you have any nasal allergies?”

### Blood samples

Two to 13 blood samples were taken per greenhouse worker (mean = 6.8 samples/person, median = 6.0 samples/person). A total of 217 blood samples were collected during the study period. Blood samples were drawn Monday morning after the work had started and the following Thursday at noon. Seven times a worker was present on a Monday but not on a Thursday, and six times a worker was present on a Thursday but not on a Monday, and these samples are included in the 217 blood samples. Blood sampling was only performed if the worker was at work. Only one blood sample was taken from each of the controls. Blood samples were drawn into plain vacutainer tubes (BD vacutainer gold top serum separator), inverted five times, left to clot for minimum 60 min and centrifuged at 3000 rpm for 15 min to separate serum. Serum was frozen at −80 °C until analysed.

### Exposure assessment

Personal exposure was measured during weeks of blood sampling, dust sampling was performed using GSP samplers (Gesamtstaubprobenahme, CIS by BGI, INC Waltham, MA, USA) on Tuesdays or Wednesdays (*n* = 115). Some of the exposure data are part of published studies [18, 26]. Sampling was performed during full work days (mean sampling time: 6 h, range: 1–8 hour). The GSP sampler samples inhalable particles (flow 3.5 l/ min). Two GSP samplers were attached to each worker’s clothing close to the breathing zone. Personal air flow was checked every hour. Each worker carried two of the described samplers, one with a polycarbonate filter (pore size 1 μm, Osmonics Inc., Minnetonka, MN, USA) for culturable counts and β-glucan and one with a teflon filter (pore size 1 μm; Millipore, Bedford, MA, USA) for endotoxin and gravimetric analysis. Each sampling day three teflon filters and one polycarbonate filter were brought as blind filters. The three teflon filters were used for gravimetric analysis and one of them subsequently as blind filter for endotoxin analysis and the polycarbonate filter as blind filter for quantification of microorganisms.

### Gravimetric analysis

The mass of the dust collected on the teflon filters was determined by weighing the filters before and after dust sampling. Before weighing, the filters were equilibrated at constant air temperature and humidity for 20–24 hours. The limit of detection when weighing the filters was 0.06 mg per filter corresponding to 0.022 mg dust/m^3^. The detection limit was calculated as three times the standard deviation of ten blanks and divided by the mean sampled volume. The data are presented as time weighted averages (TWA) in mg/m^3^ air.

### Extraction of dust

The day after each sampling materials collected on filters were extracted. The dust on the teflon filters was extracted in 6.0 ml pyrogen-free water with 0.05 % Tween 20 by orbital shaking (300 rpm) at room temperature for 60 min and centrifuging (1000 g) for 15 min. The supernatant was stored at −80 °C until it was used for the endotoxin assay. The dust collected on polycarbonate filters was extracted by placing filters in 10 ml sterile solution (0.05 % Tween 80, 0.85 % NaCl) followed by orbital shaking for 15 min (500 rpm) at room temperature.

### Quantification of endotoxin and β-glucan

The supernatants from Teflon filters were analysed (in duplicate) for endotoxin from Gram negative bacteria using the kinetic Limulus Amoebocyte Lysate test (Kinetic-QCL endotoxin kit, BioWhittaker, Walkersville, Maryland, USA) with β-glucan blocker. A standard curve obtained from an Escherichia coli O55:B5 reference endotoxin was used to determine the concentrations in terms of endotoxin units (EU) (10.0 EU ≈ 1.0 ng). The limit of detection was 0.05 EU/ml, corresponding to 0.06 EU/m^3^. The data are presented TWA in EU/m^3^ air. Endotoxin was below detection level on blind filters.

Extracts from polycarbonate filters were analysed in duplicate for β-glucan from fungi and pollen using the kinetic, chromatic Fungitic G Test (Seikaga Co., Tokyo, Japan). The triple-helix structure of the β-glucan was made water soluble by adding 0.3 M NaOH and incubating for 60 min. The detection level was 4 ng/ml. The data are presented as TWA in ng/ m^3^ air.

### Quantification of bacteria and fungi

Ten-fold dilution series of extracts from polycarbonate filters were prepared and 100 μl aliquots were placed on agar plates for quantification of bacteria and fungi. The number of inhalable fungi in polycarbonate-samples culturable on Dichloran Glycerol agar (DG18 agar, Oxoid, Basingstoke, England) at 25 °C was counted after 3 and 7 days of incubation. The numbers of inhalable bacteria were quantified after 3 and 7 days of incubation on 100 % Nutrient agar (Oxoid, Basingstoke, UK) with actidione (cycloheximide; 50 mg/l (Serva, Germany)) at 25 °C. The exposure to fungi and bacteria are expressed as TWA in colony forming units (cfu) per m^3^ air, and values were above the detection limit.

### Quantification of SAA and CRP

Serum levels of SAA were determined by Enzyme-linked Immunosorbant assay (ELISA) from Invitrogen (CA, USA) according to the manufacturer’s specifications. The range of the standard solutions was 0–600 ng/ml. Absorbance at 450 nm was measured by use of Elx808 (Biotek Instruments, Inc., Vermont, US). Sensitivity was given by the manufacturer as 4 μg/l, intra-assay variation as 4.6–7.4 % at 61.7–585.8 μg/l, and inter-assay variation as 7.0–7.8 % at 61.3–588.8 μg/l. Two controls consisting of recombinant human SAA1 in tissue culture matrix (19 mg/l and 332 mg/l) were provided with the kit and included as samples (1:1000 dilution) in all runs. The detection limit (LOD) was 4 μg/l.

Serum levels of high sensitivity CRP (CRP) were determined by ELISA from IBL International GMBH (Hamburg, Germany) according to the manufacturer’s specifications. The range of the standard solutions was 0–10 μg/ml. Absorbance at 450 nm was measured by use of Elx808 (Biotek Instruments, Inc., Vermont, US). Sensitivity was given by the manufacturer as 0.02 μg/ml, intra-assay variation as 4.1–6.9 % at 0.36–6.16 μg/ml, and inter-assay variation as 5.8–6.3 % at 0.41–6.22 μg/ml. Two controls (0.5 μg/ml and 5 μg/ml) were made in dilution buffer from Human CRP control material (code 85/506, NIBSC) and included in all runs. The LOD was 0.02 μg/ml. Concentrations below the LOD were assigned a random value between 0 and LOD extracted from a uniform distribution.

Greenhouse workers were categorized into three groups based on SAA and CRP serum levels. The cut off values used were CRP values below 1.0 mg/l for low risk for cardiovascular disease (CVD), 1.0 to 2.9 mg/l for intermediate risk for CVD and > 3.0 mg/l for high risk for CVD [12]. The SAA groups were based on measured levels in healthy subjects; SAA levels have in healthy young adults been measured to be 2.29 mg/l, in middle-aged 2.47 mg/l and in aged 3.66 mg/l [27]. As there was only one greenhouse worker in the group with lowest SAA level, we also made three new groups with cut-off-values at <10 mg/l and > 30 mg/l.

### Statistical analyses

Pearson correlation coefficient (r) on log transformed levels of SAA, CRP, endotoxin, dust, bacteria, fungi and β-glucan were calculated. Log transformed data on exposure in three different CRP and SAA groups were compared using Proc GLM.

Greenhouse workers were categorized into three groups based on SAA and CRP serum levels as described above and the bioaerosol exposures for the three groups were compared using Anova test. The associations of week day and gender on log-transformed SAA and CRP contents were estimated in mixed models accounting for correlated measures with two levels of random effects; individual and week within individual to account for correlated measures. SAA and CRP levels as an effect of week day were also compared using paired *t*-test. This was done for all workers and for workers with and without asthma. The associations of log-transformed exposure to endotoxin, dust, bacteria, fungi and β-glucan and corresponding log-transformed content of SAA and CRP were estimated in mixed models with adjustments for week day, age (<40 years or >40 years) and gender as a fixed effect and with two levels of random effects; individual and week within individual to account for correlated measures. Associations were also estimated for each gender separately; in addition we have used stepwise regression analysis with back ward elimination. Data were analysed using SAS (version 9.2).

## Results

### SAA and CRP levels

SAA and CRP levels were measured in serum samples collected from greenhouse workers and in the reference group (Table 1). SAA and CRP levels were inter-correlated for greenhouse workers (*r* = 0.62, *p* < 0.0001) and for the reference group (*r* = 0.36, *p* = 0.021, *n* = 42). There was no statistically significant difference in serum levels of SAA (*p* = 0.58) or CRP (*p* = 0.67) between greenhouse workers and the reference group (adjusted for age and gender). Exclusion of reference group members with self-reported crs or asthma symptoms had no effect (p for SAA = 0.57 and p for CRP = 0.40). When SAA levels in non-atopic greenhouse workers and non-atopic references were compared no significant difference was found (*p* = 0.83), similar no effect was seen between the two atopic groups (*p* = 0.28) and between the two groups with asthma (*p* = 0.15).

For greenhouse workers median SAA levels Monday mornings were 19.0 mg/l (average = 29.4 mg/l) and Thursday noon 14.5 mg/l (average = 26.6 mg/l); median serum CRP levels Monday morning were 1.1 mg/l (average = 1.6 mg/l) and Thursday noon 0.95 mg/l (average = 1.6 mg/l). When SAA and CRP levels were analyzed in a mixed model including only week day as factor, no significant effect of week day was found on SAA (*p* = 0.25) or CRP (*p* = 0.61) levels. Similar paired *t*-test showed no significant difference between days (for SSA *p =* 0.15, for CRP *p =* 0.86). When workers without asthma were compared separately no significant effect of week day was found for SAA (average = 25.7 versus 24.2 mg/l, *p =* 0.21) and CRP (average = 1.25 versus 1.42 mg/l, *p =* 0.50). In contrast, workers with asthma had significantly higher SAA levels Monday morning (average = 57.0 mg/l) than Thursday noon (average = 44.7 mg/l) (*p =* 0.039 in paired *t*-test), this was not seen for CRP (average = 4.3 versus 3.1 mg/l *p =* 0.23).

The individual serum SAA levels on Mondays correlated significantly with SAA levels on Thursdays (*r* = 0.62, *P* < 0.0001) within the same week. Similarly, the individual CRP levels on Mondays correlated significantly with CRP levels on Thursdays (*r* = 0.73, *P* < 0.0001) within the same week. In the following analysis we have used the average serum levels of the Monday and Thursday measurement of SAA and CRP, respectively, to correlate with the individual personal exposure levels from the same week.

### Bioaerosol exposure

Greenhouse workers were exposed to very different concentrations of bioaerosol components (Table 2). Endotoxin exposure levels correlated significantly with all other measured exposures (Table 2). Exposure of the reference group was not measured.

**Table 2:** Exposure levels and Pearson correlation coefficient (r) between exposures (*n* = 115)

	Endotoxin EU/m^3^	Dust mg/m^3^	Bacteria cfu/m^3^	Fungi cfu/m^3^	β-Glucan pg/m^3^
Average	222	0.68	3.0x10^4^	1.6x10^6^	2.8x10^5^
Median	(66)	(0.39)	(8232)	(4.3x10^4^)	(7.9 x10^4^)
Range	0.84–3100	0.042–3.22	14.7–6.4x10^5^	204–7.4x10^7^	3972–3.1x10^6^
Endotoxin, r	1	**0.31**	**0.73**	**0.69**	**0.32**
*P*-value		*<0.001*	*<0.0001*	*<0.001*	*<0.0001*
Dust, r		1	−0.0045	0.12	**0.51**
*P*-value			*0.94*	*0.066*	*<0.001*
Bacteria, r			1	**0.79**	0.13
*P*-value				*<0.0001*	*0.11*
Fungi, r				1	**0.34**
*P*-value					*<0.0001*

Values in bold are statistically significant

### Inflammatory markers and effect of bioaerosol exposure

Greenhouse workers were categorized into three groups based on SAA and CRP serum levels using cut-off values according to earlier studies [12, 27]. Endotoxin exposure was highest in the group with the highest SAA levels and in the group with middle and highest CRP levels (Table 3). Only one SAA concentration was below 3.0 mg/l; therefore we also made new categories with a cut-off value at 10 mg/l (*n* = 23 in category 1), but this did not influence the results shown in Table 3.

**Table 3:** Greenhouse workers exposure to bioaerosol components, averages and (medians) categorized by serum levels of SAA or CRP^a^

	n	Endotoxin		Dust		Bacteria		Fungi		β-glucan
		EU/m^3^		mg/m^3^		CFU/m^3^		CFU/m^3^		Pg/m^3^
SAA mg/l										
1.5–3.0	1	7.8a^c^		0.12a		1272a		4.1×10^3^a		3.2×10^3^a
(2.9)^b^										
3–30	67	184a		0.71a		3.8×10^4^a		2.4×10^6^a		2.0×10^5^a
(15.5)		(90.3)		(0.39)		(8.7×10^3^)		(4.6×10^4^)		(5.9×10^4^)
30–180	16	583b		0.75a		2.8×10^4^a		4.8×10^5^a		5.0×10^5^a
(52.2)		(241)		(0.47)		(9.0×10^3^)		(2.5×10^4^)		(8.6×10^4^)
CRP mg/l										
<1	58	156a		0.59a		1.9×10^4^a		4.6×10^5^a		2.0×10^5^a
(0.51)^a^		(49.5)		(0.30)		(6.5×10^3^)		(3.1×10^4^)		(4.7×10^4^)
1.0–3.0	41	270b		0.79a		4.2×10^4^a		3.8×10^6^a		2.4×10^5^a
(1.7)		(138)		(0.54)		(9.6×10^3^)		(5.6×10^4^)		(8.2×10^4^)
3.1–12.3	14	361b		0.60a		5.4×10^4^a		3.2×10^5^a		3.6×10^5^a
(6.2)		(136)		(0.32)		(6.9×10^3^)		(1.8×10^4^)		(8.9×10^4^)

^a^Average of measured values Monday morning and Thursday noon within the same week; one SAA in category 2 was only measured on a Thursday, while ten CRP samples were only measured Monday or Thursday.^b^Average SAA or CRP level in the category. ^c^Numbers followed by the same letter are not statistically different in an Anova test

The associations between exposure factors and average of Monday and Thursday levels of CRP or SAA, respectively, are shown in Table 4. The exposure factors were studied together and separately. When single factors were assessed, SAA levels were positively associated with endotoxin levels (*p =* 0.01), gender (women versus men, *p* = 0.047) and asthma (asthma versus no asthma, *p =* 0.0084). When all factors were included in the same model, SAA levels were positively associated with endotoxin levels (*p =* 0.026) and negatively with the levels of fungi (*p* = 0.039). In the stepwise regression analysis endotoxin, fungi and asthma were present as significant factors. All exposures correlated significantly (Table 2), and if the association between SAA level and exposure data were analyzed without endotoxin a significant effect was only seen for asthma (estimate = 0.42; *p* = 0.0084).

**Table 4:** Association between serum levels of CRP and SAA and exposure levels, age and gender

		Each factor studied separately				All factors studied in one model			
Fixed factor		SAA		CRP		SAA		CRP	
		Estimate	*p-value*	Estimate	*p-value*	Estimate	*p-value*	Estimate	*p-value*
Men and women									
Endotoxin		**0.26**	***0.011***	**0.23**	***0.016***	**0.34**	***0.026***	0.20	*0.14*
Dust		0.14	*0.46*	0.31	*0.13*	0.19	*0.39*	0.17	*0.48*
Bacteria		0.094	*0.11*	0.038	*0.54*	0.065	*0.46*	−0.066	*0.47*
Fungi		0.030	*0.59*	0.071	*0.23*	**−0.18**	***0.039***	0.031	*0.70*
β-Glucan		−0.00079	*0.99*	0.024	*0.070*	−0.013	*0.85*	−0.020	*0.73*
Asthma	Yes/no	**0.42**	***0.0084***	**0.51**	***0.011***	0.30	*0.064*	**0.48**	***0.041***
Atopy	Yes/no	0.18	*0.11*	0.12	*0.41*	0.0057	*0.95*	−0.02	*0.87*
Age group	<40 / >40 years	−0.11	*0.30*	−0.16	*0.23*	−0.018	*0.84*	−0.16	*0.22*
Gender	Women versus men	**0.24**	***0.047***	0.041	*0.78*	0.17	*0.081*	0.0085	*0.99*
Stepwise regression:^a^									
	Endotoxin	**-**	*-*	-	*-*	**0.44**	***0.0007***	**0.22**	***0.022***
	Fungi	**-**	*-*	-	*-*	**−0.16**	***0.023***	-	*-*
	Asthma	**-**	*-*	-	*-*	**0.33**	***0.016***	**0.48**	***0.015***
Men									
Endotoxin		**0.24**	***0.024***	**0.22**	***0.011***	0.27	*0.16*	0.12	*0.34*
Dust		0.30	*0.20*	**0.45**	***0.020***	0.26	*0.35*	0.23	*0.31*
Bacteria		0.090	*0.17*	0.072	*0.18*	0.092	*0.40*	−0.052	*0.55*
Fungi		0.048	*0.41*	**0.11**	***0.016***	−0.16	*0.11*	0.91	*0.20*
β-Glucan		0.061	*0.33*	**0.14**	***0.0074***	0.059	*0.48*	0.20	*0.29*
Asthma	Yes/no	**0.62**	***0.012***	**0.74**	***0.023***	0.54	*0.035*	0.60	*0.08*
Atopy	Yes/no	0.18	*0.23*	0.17	*0.38*	0.0031	*0.98*	0.06	*0.72*
Age group	<40/ >40 years	−0.083	*0.57*	−0.24	*0.14*	−0.088	*0.48*	−0.13	*0.40*
Stepwise regression:									
	Endotoxin	**-**	*-*	**-**	*-*	**0.24**	***0.024***	-	*-*
	Fungi	-	*-*	-	*-*	-	*-*	**0.12**	***0.0097***
	Asthma					**0.58**	***0.012***	**0.78**	***0.013***

Statistically significant values are in bold. ^a^ Statistically significant factors in the stepwise regression

When single factors were assessed, CRP levels were positively associated with endotoxin (*p =* 0.016) and asthma (*p =* 0.011). In the stepwise regression analysis also endotoxin was part of the best fitting model (Table 4). If the association between CRP level and exposure data were analyzed without endotoxin data no significant associations were seen.

Gender had a significant effect on SAA level and we also studied the data for the two genders separately; data are only shown for men as no significant associations were found for women. When men were studied separately a significant association was found between endotoxin exposure and SAA level and between fungi and CRP level (Table 4). If the association between CRP level and exposure data was analyzed without fungi data a significant associations was seen for endotoxin (estimate = 0.22; *p* = 0.011).

## Discussion

We found a significant association between individual exposure levels of inhalable endotoxin and serum levels of SAA and a less consistent association to CRP levels. However, SAA and CRP levels in workers exposed to bioaerosols were not different from levels found in a reference group. The reference group was selected from a population-based study on asthma and chronic rhinosinusitis. The cohort comprised a total of 365 individuals aged 15–75 years. Initially, 33 individuals without self-reported symptoms of asthma and /or crs were matched with the individual greenhouse workers to match the two groups according to sex (64 % males in both groups), age and smoking status (24 % current smokers in both groups). In addition 9 individuals with asthma and/or crs were selected (56 % males, mean age: 42.0, 44 % current smokers). The serum CRP levels of the greenhouse workers (median = 0.95 mg/l) were similar to levels previously reported for adult occupants in a wood smoke impacted community (1.00 ± 0.78 mg CRP/l) in Canada [28], for young healthy Danish individuals (geometric mean = 1.2 mg/l) [29] and Swedish middle-aged individuals (median = 0.74 mg/l) [27]. In this study the median serum level of SAA of greenhouse workers was 15.2 mg/l (average = 28.3 mg/l). This is higher than SAA median levels in healthy young adults: 2.29 mg/l, in middle-aged 2.47 mg/l and in aged 3.66 mg/l healthy Swedish individuals [27]. The median SAA level in greenhouse workers without atopy and asthma was 11.1 mg/l (median = 26.5 mg/l), which is also higher than median levels in healthy Swedish individuals. This may be due to endotoxin exposure; on the other hand the reference group also had high SAA levels. Greenhouse workers with asthma had significantly higher SAA levels than colleagues without asthma; this is not surprising as SAA is a marker of both asthma and atherogenesis [30]. The found associations between endotoxin exposure levels and SAA and CRP levels in serum may suggest a causal association even though the levels were not increased compared to the reference group. In another study, the prevalence of self-reported respiratory symptoms tended to be higher among greenhouse workers compared to controls [19]. Inhalation of LPS has previously been shown to dose-dependently increase CRP levels in healthy subjects [31]. Thus, inhalation of 5 μg and 50 μg of LPS by human volunteers induced a dose-dependent increase in CRP levels [31]. Inhalation of 5 μg LPS (corresponding to about 50 000 EU units) increased CRP to ~ 4 μg/ml, whereas inhalation of 50 μg (500 000 EU units) increased CRP levels to ~30 μg/ml. SAA was not assessed in that study. In mice, inhalation of LPS induced a strong transcriptional activation of *Saa1* and *Saa3* (both encoding SAA isoforms) in lung tissue [10]. In the current study, workers were exposed to between 0.84 and 3100 EU/m^3^ (median = 66 EU/m^3^). Assuming that humans inhale 0.45 m^3^/h, this would correspond to inhalation of between 3.0 and 11160 EU/work day (median = 238 EU/work day) during an 8-hours working day.

We used the study design with blood sampling Monday morning and Thursday noon in the same week as one could expect an increase in inflammatory marker levels during the work week or during a work day. However, we did not find significant differences in SAA and CRP levels on Monday versus Thursday measurements. There are several possible explanations. Some workers may have been exposed during activities in the weekend. Secondly, inhaled endotoxins may be cleared slowly from the lungs, resulting in prolonged exposure. Lastly, it takes approximately 7 days for SAA and CRP levels to return to baseline levels after an acute phase response [6] and thus, serum levels of acute phase proteins on Mondays may reflect exposure during the previous week. A study with bioaerosol exposed biofuel workers showed no increase in levels of the inflammatory markers IL-1β and IL-8 from Monday morning to Thursday noon [32]. In contrast, a study with bioaerosol exposed waste handlers showed increased levels of inflammatory markers (neutrophils and IL-8 levels) from Monday morning to Thursday [33]. When greenhouse workers with asthma were studied separately a significant effect of week day was found – but with higher SAA levels on Mondays than on Thursdays. We do not know whether this is related to the reduced mucociliary clearance in people with asthma [34] leading to a longer exposure period, or alternatively that asthma patients Monday morning react faster to endotoxin exposure.

SAA level was significantly and negatively associated with exposure to fungi, but CRP level was positively associated with men’s exposure to fungi (Table 4). For waste handlers exposed to bioaerosols, inflammatory responses were related significantly to levels of exposure to endotoxin, but not to fungi and bacteria [33]. We measured large differences in the exposure levels to fungi (between 204–7.4x10^7^ cfu/m^3^); large differences have also been found in other greenhouse studies (between bd and 7.4x10^7^ cfu/m^3^) [35]. The median (4.3x10^4^ cfu/m^3^), but not the maximum (7.4x10^7^ cfu/m^3^) fungal exposure of greenhouse workers was lower than exposures of workers developing Organic Dust Toxic syndrome (ODTS) (2-8x10^6^ cfu/m^3^) [36], but higher than concentrations measured using stationary samplers in herb processing plants (mean = 10^4^ cfu/m^3^) [37]. The results of occupational exposure to fungi and related health effects are divergent [2, 33], and we suggest that different fungal species present in the different exposures, also within an environment, may affect the biological response, and it may be relevant to distinguish between fungal species or fungal inflammogens. A recent study indicates that not only the concentration of microorganisms and microbial enzymes, but also the microbial composition is different in organic dust causing airway symptoms than in reference dust [38]. Fungi contain β-glucan in their cell walls and β-glucan is an inflammogen [39]; in this study divergent results were also found for β-glucan. We have used two approached by quantifying both living microorganisms and microbial components. The two components endotoxin and β-glucan are well described inflammogens and represent both cultivable and non-cultivable bacteria and fungi. We have chosen to measure cultivable fungi because germinable or living spores are more inflammogene than non-germinable or dead spores [40, 41].

Serum levels of the acute phase proteins SAA and CRP are known risk factors for cardiovascular disease in prospective studies [13, 42]. Conditions that induce acute phase response are in general associated with increased risk of cardiovascular disease [43–45]. Furthermore, experimental evidence from animal studies suggests a causal relation between SAA and atherosclerosis [5]. Thus, occupational exposures that lead to increased acute phase response are likely associated with risk of cardiovascular disease [46] especially since the exposure may occur continuously during a 40 year work life. The acute phase response is a general defense response, and therefore, many different factors including lifestyle factors influence SAA and CRP levels. Use of anti-inflammatory medicine such as aspirin and statin reduces inflammation and acute phase response, whereas smoking and high BMI is associated with increased levels of both SAA and CRP [47, 48]. We did not control for these factors in the present study, but the comparison group was matched on age, gender and smoking.

The measured personal endotoxin exposure levels in this study were very variable (from 0.84 to 3100 EU/m^3^) and were similar to previously measured exposure levels for greenhouse workers [18, 19]. The endotoxin exposure levels were lower than the exposure levels of grass seed workers developing ODTS [36] and of grain farmers [49]. Since personal exposure to inhalable endotoxin is associated with serum levels of acute phase proteins, we suggest reducing occupational exposure to endotoxin. We have previously identified factors affecting exposure to bioaerosols in greenhouses and have shown that it is possible to reduce exposure to bioaerosols [18, 26]. Thus endotoxin exposure was reduced significantly by cleaning out old plants before they were dried out [18]. In another working environment with high endotoxin exposure, exposure cessation has caused lung function improvement after a year, but mainly for the lowest exposed workers [50].

## Conclusion

In conclusion, serum levels of the acute phase proteins CRP and SAA were not higher in greenhouse workers than in a reference group. However, occupational exposure to endotoxin was positively associated with serum levels of the acute phase protein SAA and less consistently with CRP. Increased inflammation is linked to airway diseases and increased risk of cardiovascular disease and preventive measures to reduce endotoxin exposure may therefore be beneficial.

## Abbreviation

Bd: Below detection limit
Cfu: Colony forming units
CRP: C-reactive protein
CRS: Chronic rhinosinusitis
ELISA: Enzyme-linked Immunosorbant assay
EU: Endotoxin units
GSP: Gesamtstaubprobenahme, an aerosol sampler
LOD: Limit of detection
ODTS: Organic Dust Toxic Syndrome
r: Pearson correlation coefficient
SAA: Serum Amyloid A
TWA: Time weighted average
